# Evidence for a bimodal distribution of *Escherichia coli *doubling times below a threshold initial cell concentration

**DOI:** 10.1186/1471-2180-10-207

**Published:** 2010-08-02

**Authors:** Peter L Irwin, Ly-Huong T Nguyen, George C Paoli, Chin-Yi Chen

**Affiliations:** 1 Molecular Characterization of Foodborne Pathogens Research Unit, Eastern Regional Research Center, Agricultural Research Service, U. S. Department of Agriculture, 600 East Mermaid Lane, Wyndmoor, PA 19038 USA

## Abstract

**Background:**

In the process of developing a microplate-based growth assay, we discovered that our test organism, a native *E. coli *isolate, displayed very uniform doubling times (τ) only up to a certain threshold cell density. Below this cell concentration (≤ 100 -1,000 CFU mL^-1 ^; ≤ 27-270 CFU well^-1^) we observed an obvious increase in the τ scatter.

**Results:**

Working with a food-borne *E. coli *isolate we found that τ values derived from two different microtiter platereader-based techniques (i.e., optical density with growth time {=OD[t]} fit to the sigmoidal Boltzmann equation or time to calculated 1/2-maximal OD {=t_m_} as a function of initial cell density {=t_m_[C_I_]}) were in excellent agreement with the same parameter acquired from total aerobic plate counting. Thus, using either Luria-Bertani (LB) or defined (MM) media at 37°C, τ ranged between 17-18 (LB) or 51-54 (MM) min. Making use of such OD[t] data we collected many observations of τ as a function of manifold initial or starting cell concentrations (C_I_). We noticed that τ appeared to be distributed in two populations (bimodal) at low C_I_. When C_I _≤100 CFU mL^-1 ^(stationary phase cells in LB), we found that about 48% of the observed τ values were normally distributed around a mean (μ_τ1_) of 18 ± 0.68 min (± σ_τ1_) and 52% with μ_τ2 _= 20 ± 2.5 min (n = 479). However, at higher starting cell densities (C_I_>100 CFU mL^-1^), the τ values were distributed unimodally (μ_τ _= 18 ± 0.71 min; n = 174). Inclusion of a small amount of ethyl acetate to the LB caused a collapse of the bimodal to a unimodal form. Comparable bimodal τ distribution results were also observed using *E. coli *cells diluted from mid-log phase cultures. Similar results were also obtained when using either an *E. coli *O157:H7 or a *Citrobacter *strain. When sterile-filtered LB supernatants, which formerly contained relatively low concentrations of bacteria(1,000-10,000 CFU mL^-1^), were employed as a diluent, there was an evident shift of the two populations towards each other but the bimodal effect was still apparent using either stationary or log phase cells.

**Conclusion:**

These data argue that there is a dependence of growth rate on starting cell density.

## Background

Understanding the behavior of bacterial growth parameters (duration of lag phase, specific growth rate, and maximum cell density in stationary phase) under various environmental conditions is of some interest [1]. In particular, knowledge about growth parameter population distributions is needed in order to make better predictions about the growth of pathogens and spoilage organisms in food [1-3]. In fact, probability-based methods, such as microbial risk assessment [1], have to take into account the distribution of kinetic parameters in a population of cells [4]. There is a paucity of growth parameter distribution data because of the large number of data points required to obtain such results. The utilization of traditional microbiological enumeration methods (e.g., total aerobic plate count or TAPC) for such a body of work is daunting. For this reason various methods have been developed which enable more rapid observations related to one, or more, growth parameters. Recently, growth parameter distribution characterization has mainly focused on the duration of lag phase [4-8]. For instance, Guillier and co-workers studied the effects of various stress factors (temperature, starvation, salt concentration, etc.) on individual cell-based detection times in *Listeria monocytogenes *[5,6]. Additionally, reporting on improved methods, various workers [4,7,8] have presented frequency distribution information concerning lag phase duration of individual bacterial cells (*Escherichia coli*, *L. monocytogenes*, and *Pseudomonas aeruginosa*) on solid media. However, similar population-based information on specific growth rate is lacking.

The findings presented herein developed from work associated with the attachment of various Gram-negative bacteria to anti-*Salmonella *and anti-*E. coli *O157 immunomagnetic beads or IMBs [9-11]. For these IMB investigations microplate (OD-based) MPN methods were utilized because of the low limits of bacterial detection [12,13] necessary to characterize the non-specific attachment of background food organisms to various capture surfaces. Because of large inter-bacterial strain variability in the time requisite to reach a measurable level of turbidity, we found it necessary to characterize the growth rate and apparent lag time (time to 1/2-maximal OD or t_m_) [12] of certain problematic organisms. Toward this end we began a routine investigation into the best microplate reader method to determine doubling time (τ). However, while performing this work we noticed that our test organism, a native *E. coli *isolate which non-specifically adheres to certain IMBs [11], seemed to display very uniform τ values only up to a certain threshold initial or starting cell density (C_I_) beyond which we observed an obvious increase in the scatter. A larger number of observations were then made after various physiological perturbations (media used, growth phase, etc.) which have lead to the results discussed in this report.

## Results and Discussion

### Doubling Times from both TAPC and Microplate Observations

Table 1 shows analysis of variance data for τ calculated as described in the Methods Section from Optical Density with time (= OD[t]; **Eq. 1**) data, t_m _as a function of C_I _(= t_m_[C_I_]; **Eq. 6**), and total aerobic plate count with time (= TAPC[t]) on two different media at 37°C (C_I _> 1,000 CFU mL^-1^). These results indicate that doubling times derived from the aforementioned microplate techniques (i.e., OD[t] and t_m_[C_I_]) were in excellent agreement with τ values acquired from TAPC when using either Luria-Bertani (LB) or a defined minimal medium (MM) at 37°C. In these experiments τ varied 17 to 18 min (LB) or 51 to 54 min (MM) depending on media. The within-medium variation was not significant at even a 0.1 level (i.e., the probabilities of  > 3.43 was 0.136 and  >0.886 was 0.480). These results show that both microplate-based methods for measuring τ are equivalent to τ derived from TAPC. For low initial cell concentrations, the OD[t] method, as described in the Methods section, is obviously superior to t_m_[C_I_] since it makes no assumption about concentration dependence. However, for routine growth studies (e.g., antibiotic resistance) at a relatively high C_I _the t_m_[Φ_I_] method (**Eq. 5**, Methods Section; Φ_I _is the dilution factor used to make each C_I_) for obtaining τ is preferable since t_m _is easy to obtain without curve fitting albeit several dilutions need to be used.

**Table 1:** Comparison of doubling time (= **τ**) observations based on total aerobic plate counting with growth time (= TAPC[t]), time to 1/2-maximal OD with growth time (= t_m_[t]), and optical density at 590 nm with time (= OD[t]) in either LB broth or MM at 37°C.

Method	τ (min) -- LB				
			
	Exp. 1	2	3	average	F_2,4_
					
TAPC[t]	18.6	17.3	18.1	18.0	3.43
t_m_[Φ_i_]	17.1	17.4	16.8	17.1	*P >*0.1
OD[t]	17.9	17.9	17.7	17.8	
					

**Method**	**τ (min) --MM**				
			
	**Exp. 1**	**2**	**3**	**average**	**F2,4**
					
TAPC[t]	52.7	50.1	51.9	51.6	0.886
t_m_[Φ_i_]	50.8	59.9	52.1	54.3	*P >>*0.1
OD[t]	50.1	53.8	49.4	51.1	

**Figure 1:**
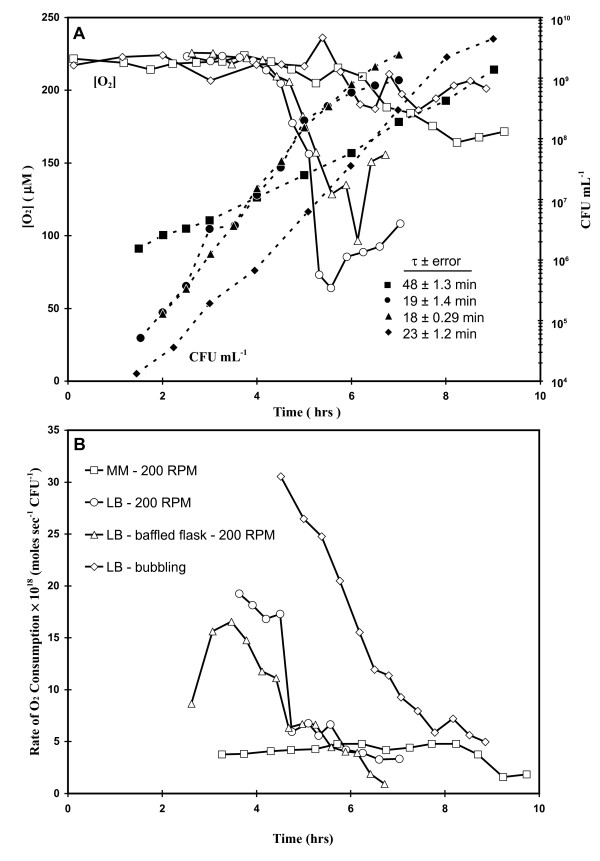
**Steady state O_2 _([O_2_]: Fig 1A, open symbols), O_2 _consumption rates (normalized to TAPC: Fig 1B) and *E. coli *cell growth (Fig 1A, closed symbols) as a function of growth time at 37°C in various media**. Culture volume = 100 mL minimal defined medium (MM) or Luria-Bertani (LB) broth in a 250 mL normal or baffled Erlenmeyer flasks; 200 RPM agitation: squares = MM, normal flask; circles = LB, normal flask; triangles = LB, baffled flask; diamonds = LB, air bubbled in addition to shaking.

The agreement between the *E. coli *τ from TAPC and microplate methods was somewhat unexpected inasmuch as solution agitation (i.e., oxygenation) of the media in each plate's wells would be less than that for solution agitation in either normal or baffled flasks which were used for the TAPC comparisons. However, we found (Fig. 1A, open symbols) that [O_2_] levels in even highly agitated liquid *E. coli *cultures at 37°C dropped as much as 72% (LB, normal flask) with 200 RPM shaking while they were consuming approximately {4-6} × 10^-18 ^moles O_2 _sec^-1 ^CFU^-1 ^(Fig. 1B). Even the baffled flask culture showed a drop in [O_2_] of 40-57%. Simultaneously, no cultures (Fig. 1A, closed symbols) showed any perturbations in τ (~ 18 min); the 23 min τ seen with bubbling is probably greater due to evaporative cooling of the medium. Due to differences in both solution mixing and surface area-to-volume ratio, the [O_2_] levels in microplate wells must be even lower than flask cultures at equivalent cell densities. Fig. 1 demonstrates that even at the lowest [O_2_], the rates of growth were unaffected. Clearly, being a facultative anaerobe, *E. coli *is able to rapidly adjust to different levels of O_2 _with no apparent change in its specific growth rate, although the maximum cell density in stationary phase is usually greater in highly oxygenated samples by up to an order of magnitude.

### Effect of Initial or Starting CFU Concentration on τ

**Figure 2:**
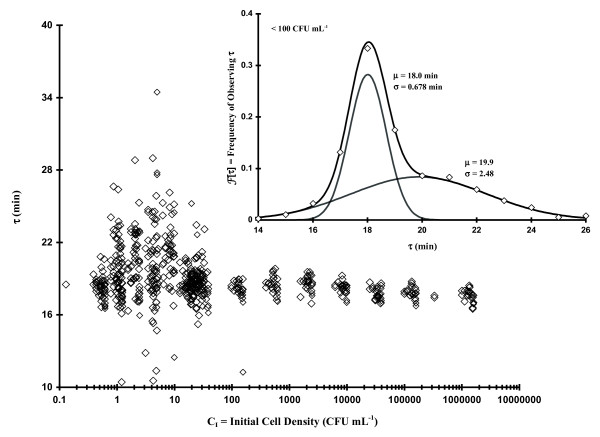
**Plot of 653 observations of τ as a function of initial cell concentration (C_I_; dilute stationary phase *E. coli *cells)**. Inset Figure: **Frequency of occurrence of various values of **τ **(C_I _< 100 CFU mL^-1^) fit to Eq. 7**.

While performing studies related to comparing various assays for determining growth rate (Table 1), we noticed that our test organism, a nonpathogenic avian *E. coli *isolate, seemed to display uniform OD[t]-based τ values up to a threshold C_I_, at which point there was an obvious increase in the observed τ scatter (Fig. 2). The main graph in Fig. 2 represents 653 measurements of τ derived from OD[t] data using **Eq. 1 **(Methods Section) plotted as a function of C_I _(diluted from stationary phase cells). When C_I _> ca. 100 CFU mL^-1^, τ was narrowly Gaussian-distributed (i.e., a unimodal distribution) with a total spread of ca. 16 to 20 min (n = 174 observations: μ_τ _± σ_τ _= 17.6 ± 0.708 min using a single Gaussian distribution function: i.e., **Eq. 7 **with α = 1 and β = 0; Methods Section). However, when C_I _< ca. 100 CFU mL^-1 ^there was a clear broadening in the range of observed τ values (ca. 10 to 34 min). At such low concentrations the CFUs per well should vary between 1 and 10 whereupon 44% of the wells should have 1 (± 1) CFU per well, 14% with 2 (± 1.4) CFUs per well, 8% with 3 (± 1.7) per well, 6% with 4 (± 2) per well, and 3% with between 5 (± 2.2) and10 (± 3.2) CFUs per well (assuming a Poisson distribution of CFU counts). The inset graph in Fig. 2 shows frequency of occurrence for all values of τ, which occur in the region of greatest scatter (C_I_< 100 CFU mL^-1^), with the best fit bimodal Gaussian distribution (**Eq. 7**) represented by the solid, black curve. The least squares bimodal distribution curve fit contains a narrow component (α ~0.48; μ_τ1 _± σ_τ1 _= 18.0 ± 0.678 min) similar to the high cell concentration-associated unimodal distribution. Based upon area, there was also a nearly equivalent broad component (β ~ 0.52; μ_τ2 _± σ_τ2 _= 19.9 ± 2.48 min). Each constituent of this bimodal distribution is shown as a solid, grey curve.

**Figure 3:**
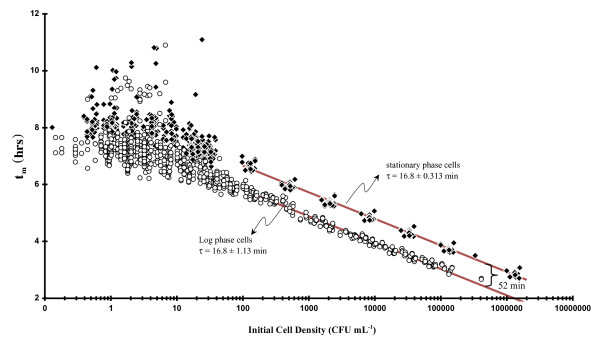
**Dependence of numerous t_m _observations on initial bacterial cell concentration (C_I_; Eq. 6) as a function of growth phase of the initial inoculum (log or stationary phase)**: circles = Log phase cells (τ = 16.8 ± 1.13 min); diamonds = stationary phase cells (τ = 16.8 ± 0.313 min).

A similar increase in another growth parameter's scatter was also observed with the t_m_[C_I_]data at low C_I _(Fig. 3) whereupon we saw that t_m _values changed in a predictable way (e.g.,|∂t_m_/∂Log_2_C_I_| = τ) up to CI ~ 100 - 1,000 CFU mL^-1 ^at which point they began to show an obvious large deviation in t_m _(between 6 and 11 hrs). These perturbations in t_m_ at low C_I _confirm the τ observations because t_m _is modulated, at least in part, by τ (**Eqs. 5-6**: all t_m _& T-based equations are developed in the Methods Section) and therefore large deviations in τ (Fig. 2) should result in increased scatter in t_m _as well. Working with stressed *Listeria monocytogenes*, Guillier and coworkers [5] observed numerous values of a lag time-related growth parameter with a similar asymmetric distribution pattern. Measuring the time of the first cell division in *E. coli *using a microscopic method, which should provide the true value of lag time, Niven and co-workers [8] were ableto make numerous observations (n = 434) which showed a very broad (μ_T_~ 184 ± 45 min; our calculation assuming a unimodal distribution) asymmetric distribution. Asymmetry might be interpreted as weakly bimodal. Other workers [4] using a different method of observation showed that the distribution of individual times to the first cell division varied greatly based on salt concentration. In fact, at high salt concentrations, the distribution pattern appeared distinctly bimodal. However, in earlier work [7], such asymmetric population distributions were interpreted as being Gamma-distributed.

**Figure 4:**
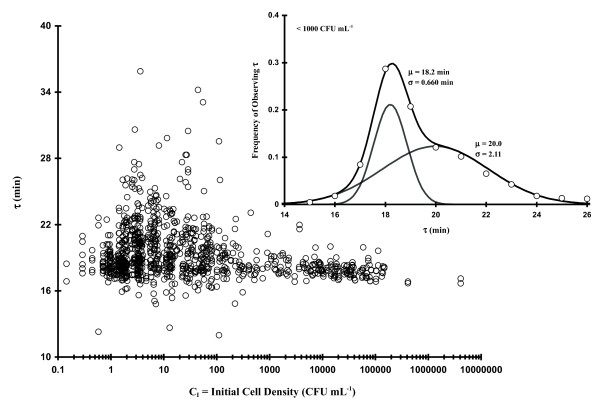
**Plot of 987 observations of τ as a function of initial cell concentration (C_I_; diluted log phase *E. coli *cells)**. Inset Figure: **Frequency of occurrence of various values of **τ **(C_I _< 1000 CFU mL^-1^) fit to Eq. 7**.

The experiments represented in Fig. 2 were repeated using mid-log phase-associated cells as described in the Experimental section and we saw qualitatively similar results (Fig. 4). The main graph in Fig. 4 represents 987 OD[t] observations with the calculated values of τ plotted as a function of C_I_. At C_I_s > ca. 1,000 CFU mL^-1 ^the average τ was unimodally-distributed with a maximum spread of ca. 17 to 22 min (159 observations; μ_τ _± σ_τ _= 17.9 ± 0.645 min). Similar to the stationary phase-based cells, we see that as C_I _was decreased (C_I _≤ 200 CFU mL^-1 ^or ≤ 54 ± 7.3 CFU/well), a striking increase occurred in the scatter of τ (spread between 12 and 36 min). The frequency of occurrence of all log phase-based τ values (C_I _< 1,000 CFU mL^-1^) are displayed in the inset graph of Fig. 4 (α ~ 0.35; μ_τ1 _± σ_τ1 _= 18.2 ± 0.660 min; β ~ 0.65; μ_τ2 _± σ_τ1 _= 20.0 ± 2.11 min).

It is important to keep in mind throughout this work that by the time we begin to observe an increase in OD (and therefore measure τ via **Eq. 1**), somewhere between 2 and 20 doublings will have occurred. This fact implies that the values we observe are somehow modulated based upon initial conditions. It should also be noted that low bacterial C_I_s (i.e., ≤ 5 CFU mL^-1^) would result in at least some single CFU occurrences per well (i.e., the average probability of observing 1 CFU per well should be about 32.0 ± 6.65%) at which point the first few events of cell division could modulate characteristics of both τ and true microbiological lag time (T). Thus, some of the increase in τ and T scatter we observe at low C_I _could result from the random selection of isolates with particularly slow growth rates which would otherwise be masked by other isolates in the media with faster rates. However, arguing against such a stochastically-based explanation is the fact that a significant fraction of the scatter in τ (Figs. 2 and 4) occurs between C_I _= 10-100 CFU mL^-1 ^whereupon the probability of observing 1 CFU per well only ranges from 18.1 to ca. 0%. Under these conditions the random selection of one particular τ-component would be overwhelmed by the sheer number of other cells present. At slightly higher concentrations (e.g., 2 or 3 CFUs per well), any well which has 2 or 3 cells with τ values differing more than about 4 or 5 min would be obvious in the ∂OD[t]/∂t curves as additional peaks. Nevertheless, we just don't observe such behavior at these low C_I_s. What we do observe are relatively uniform, monotypic growth curves (examples in Methods Section) indicative of one component (or, if more than one, the Δτs are small). The fact that we see much greater τ-based scatter at a relatively large threshold C_I _argues that there is some other controlling factor in determining such binomial-based population growth rates.

**Figure 5:**
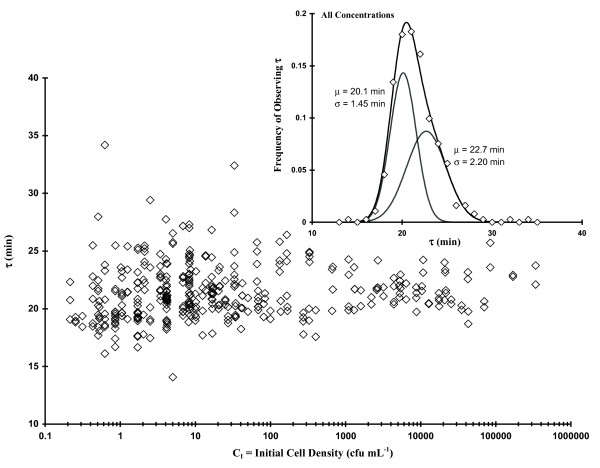
**Plot of 372 observations of τ as a function of initial cell concentration (C_I_; LB with 75 mM EA-diluted log phase generic *E. coli *cells)**. Inset Figure: **Frequency of occurrence of various values of τ (C_I _= all CFU mL^-1^) fit to Eq. 7.**

In order to determine if the apparent C_I _effect on τ was only associated with our native *E. coli *strain, we tested two other bacterial strains (*E. coli *O157:H7 and *Citrobacter*). Table 2 summarizes τ frequency distribution parameters (**Eq. 7**, Methods Section) from the experiments represented in Figs. 2 and 4 as well as results concerning mid-log phase *E. coli *O157:H7 and *Citrobacter *in LB, *E. coli *in MM or LB with 75 mM ethyl acetate (EA; solvent for N-acyl homoserine lactones). The stationary or log phase-based generic *E. coli *or *E. coli *O157:H7 growth data in LB gave similar results: for the narrower portion of the bimodal Gaussian distribution, the population mean τ values (μ_τ1_) varied only 18.0 to 18.5 min (σ_τ1 _0.401 to 0.678); the broader part of the distribution was also very similar (μ_τ2 _= 19.9 to 20.1 min; σ_τ2 _2.01 to 2.48). Utilizing MM rather than LB with generic *E. coli *cells from log phase cultures, we saw that the τ distribution on initial cell concentration remained as apparent as the phenomenon in LB (μ_τ1 _± σ_τ1 _= 51.1 ± 1.75 min; μ_τ2 _± σ_τ2 _= 56.9 ± 8.32 min), which is consistent with other work (Table 1). The Gram negative bacterium *Citrobacter *(Table 2), which was also grown in LB with cells from log phase cultures, had relatively large doubling times but displayed a clear bimodal distribution in τ at low cell densities (α = 0.6, μ_τ1 _± σ_τ1 _= 42.5 ± 3.75 min; β = 0.4, μ_τ2 _± σ_τ2 _= 50.7 ± 6.5 min) similar to previous observations. However, the ethyl acetate set of experiments (LB with 75 mM EA) with *E. coli*, which were performed as a positive control for testing various N-acyl homoserine lactones (AHL; in Gram-negative bacteria AHL is one of two major types of quorum sensing compounds believed to regulate various aspects of bacterial physiology depending upon population size), showed that EA nearly collapsed the bimodal distribution (Fig. 5) to a unimodal form as a result. We observed that α dropped to 0.15 from an LB average of 0.41 (± 0.066), μ_τ1 _shifted upward 1.4 min, and σ_τ1 _broadened by 0.339 min. This result argues for a physiological basis for the increased τ scatter at C_I _below 100 (stationary phase Fig. 2) to 1,000 (log phase Fig. 4) CFU mL^-1^. Because of the relatively large effect of solvent alone, the AHL experiments were not performed.

**Table 2:** Comparison of doubling time distribution parameters (Eq. 1) for *E. coli*, *E. coli *O157:H7, and *Citrobacter *in LB, LB + ethyl acetate (EA, 75 mM), or MM at 37°C; S = Stationary phase, L = Log Phase.

		**C_I _≤ 100 CFU mL^-1 ^**					**C_I _≥ 1000 CFU mL^-1^**
			
**Organism (phase)**	**Medium LB**	**α**	**μ_τ 1 _± σ_τ1_**	**β**	**μ_τ2 _± σ_τ2_**	**Δμ_τ_**	**μ_τ _± σ_τ_**
							
*E. coli *(S)	LB	0.48	18.0 ± 0.678	0.52	19.9 ± 2.48	1.87	17.6 ± 0.708
*E. coli *(L)	LB	0.35	18.2 ± 0.660	0.65	20.0 ± 2.11	1.79	17.9 ± 0.645
*E. coli *(L)	LB+EA	0.15	19.6 ± 0.999	0.85	21.7 ± 2.25	2.13	21.4 ± 2.06
*E. coli *(L)	MM	0.30	51.1 ± 1.75	0.70	56.9 ± 8.32	5.77	52.0 ± 2.09
							
*E. coli *O157:H7 (L)	LB	0.40	18.5 ± 0.401	0.60	20.1 ± 2.01	1.60	18.1 ± 0.438
							
*Citrobacter *(L)	LB	0.6	42.5 ± 3.75	0.40	50.7 ± 6.50	8.24	42.4 ± 3.72

**Figure 6:**
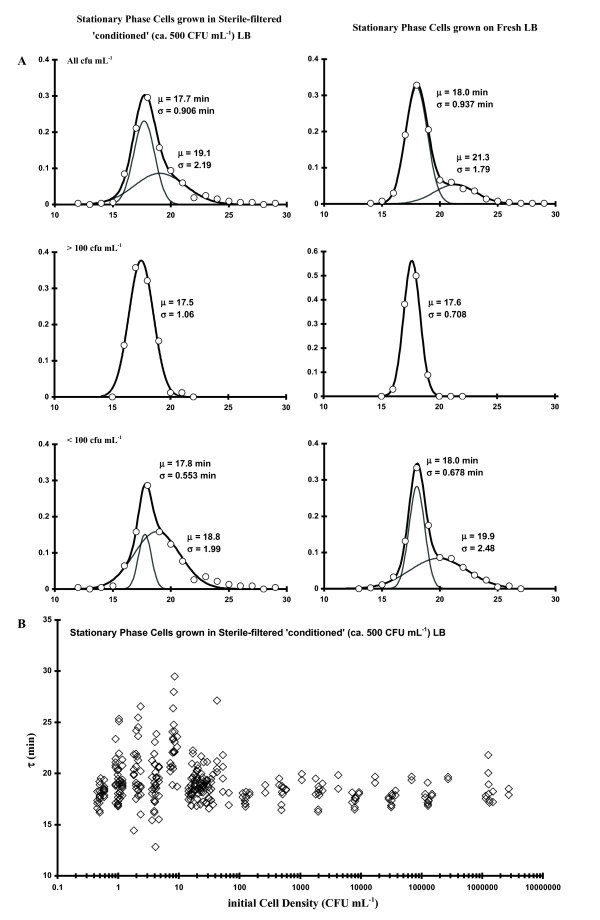
**Frequency of occurrence of various values of τ (all C_I_; C_I _> 100; C_I _< 100 CFU mL^-1^, from top to bottom)**. Left-hand side plots: stationary phase cells diluted with and grown in sterile-filtered 'conditioned' LB. Right-hand side plots: stationary phase cells diluted with and grown in LB.

**Figure 7:**
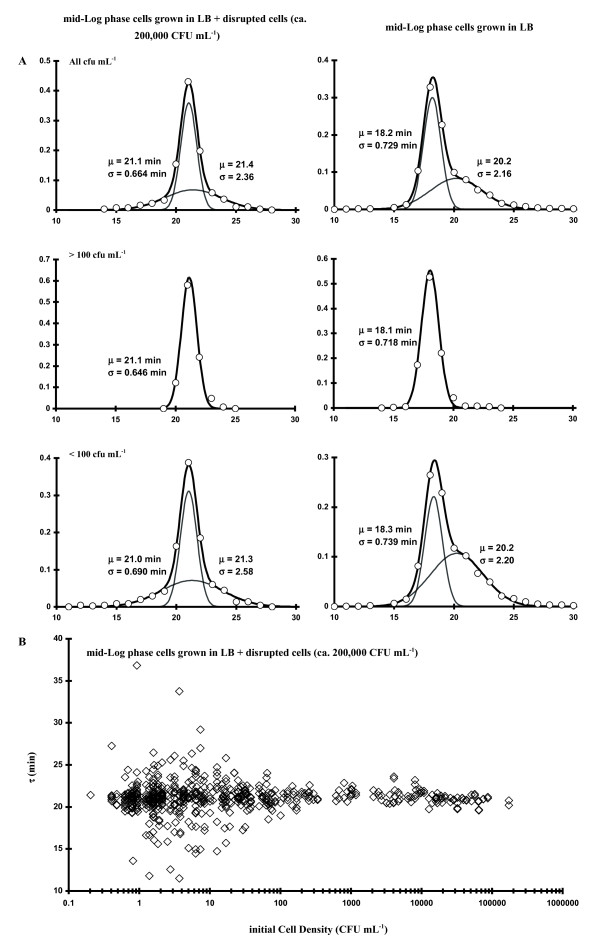
**A: Frequency of occurrence of various values of τ (all C_I_; C_I _> 100; C_I _< 100 CFU mL^-1^, from top to bottom)**. Left-hand side plots: mid-log phase cells diluted with and grown in LB with ~2×10^5^ CFU mL^-1 ^of disrupted cells LB. Right-hand side plots: mid-Log phase cells diluted with and grown in LB. **B:Plot of 572 observations of **τ **as a function of initial cell concentration (C_I_; diluted with and grown in LB with ~ 2×10^5 ^CFU mL^-1 ^of disrupted *E. coli *cells LB)**.

Since there was an obvious dependence of τ on C_I_, we were interested in determining if the bimodal effect could be reversed by growth in sterile-filtered LB media, which formerly contained the same bacterial isolate (i.e., 'conditioned' media), thus testing to see if an extracellular molecule modulated the bimodal distribution effect (i.e., related to quorum sensing). In one set of experiments (stationary phase inoculum) the LB diluent was made as follows: 37°C LB was inoculated with stationary phase *E. coli *cells and grown several hrs at 37°C (up to ca. 500 CFU mL^-1^) followed by sterile filtering (2 μm) after centrifugation. These observations are plotted adjacent to control data (Fig. 2) in Fig. 6. A second (log phase cells) experiment was also performed (after harvesting an inoculum for the experiment, the mid-log phase LB medium was centrifuged, sterile-filtered and 20 μL added to each well for the growth experiment), with the results shown in Table 3. Both experiments showed that there was a shift in the low C_I _bimodal populations (Δμ_τ _from 1.8 to 1 min) but the bimodal effect was still apparent. The treatments depicted in Fig. 6 also clearly conceptualize the line broadening of the narrow distribution component, the relative decrease in α in the bimodal population, as well as the shift of the two bimodal components towards each other. Thus, some component exists in the media which somewhat modulates the growth process. Lastly, when approximately 2 × 10^5 ^sonicated/heat-killed cells mL^-1 ^in fresh LB were utilized as the diluent but with the starting innocula taken from a log phase culture, the effect was to induce the narrow component's average τ to shift to that of the broad component (e.g., μ_τ1 _~ μ_τ2_, Δ μ~ 0; Fig. 7A, left hand side of plots). Fig. 7B shows τ data plotted as a function of C_I _and clearly shows the initial concentration effect of τ scatter below 100 CFU mL^-1^. These results also argue for a physiological basis for the increased τ scatter at relatively low C_I _(Figs. 2 and 4).

**Table 3:** Comparison of doubling time distribution parameters (Eq. 1) for *E. coli *in LB, or in LB with sonicated and heat-killed cells at 37°C; S = stationary phase, L = Log phase.

	**C_I _≤ 100 CFU mL^-1^**					**C_I _≥ 1000 CFU mL^-1^**
		
**Organism (phase)**	**α**	**μ_τ1 _± σ_τ1_**	**β**	**μ_τ2 _± σ_τ2_**	**Δμ_τ_**	**μ_τ _± σ_τ_**
						
Control LB (S)	0.48	18.0 ± 0.678	0.52	19.9 ± 2.48	1.87	17.6 ± 0.708
Conditioned LB (S)	0.21	17.8 ± 0.553	0.79	18.8 ± 1.99	1.03	17.5 ± 1.06
Control LB (L)	0.35	18.2 ± 0.660	0.65	20.0 ± 2.11	1.79	17.9 ± 0.645
Conditioned LB (L)	0.31	19.1 ± 0.627	0.69	20.1 ± 2.10	0.994	18.9 ± 0.700
Sonicated, Heat-killed Cells in LB (L)	0.54	21.0 ± 0.690	0.46	21.3 ± 2.58	0.300	21.1 ± 0.646

## Conclusion

Working with a native, food-borne *E. coli *isolate grown in either LB or MM, we found that microplate-based doubling times were bimodally distributed at low cell densities using either log or stationary phase cells as an initial inoculum. Qualitatively identical results were obtained for an *E. coli *O157:H7 and *Citrobacter *strain. When sterile-filtered 'conditioned' LB media (formerly contained relatively low concentrations of bacteria or sonicated/heat-killed cells) were employed as a diluent, there were apparent shifts in the two (narrow and broad) populations but the bimodal effect was still evident. However, the bimodal response was almost completely reversed when the growth media contained a small amount of ethyl acetate.

The clear doubling time-cell concentration dependency shown in these results might indicate that bacteria exude a labile biochemical which controls τ, or a need for cell-to-cell physical contact. The latter proposal seems unlikely inasmuch as the probability of random contact would be small at such low cell densities (C_I _~ 100-1,000 CFU mL^-1^). Perhaps this anomalous bimodal distribution of doubling times is related to the recently proposed phenotypic switching [14,15] which describes programmed variability in certain bacterial populations.

## Methods

### General

*Escherichia coli *(non-pathogenic chicken isolate) [11], *E. coli *O157:H7 (CDC isolate B1409), and *Citrobacter freundii *(non-pathogenic poultry isolate; identification based on 16 S rDNA analysis) [16] were cultured using LB (Difco) or MM (60 mM K_2_HPO_4_, 33 mM KH_2_PO_4_, 8 mM (NH_4_)_2_SO_4_, 2 mM C_6_H_5_O_7_Na_3 _[Na Citrate], 550 μM MgSO_4_, 14 μM C_12_H_18_C_l2_Na_4_OS [Thiamine•HCl], 12 mM C_6_H_12_O_6 _[glucose], pH 6.8). Liquid cultures were incubated with shaking (200 RPM) at 37°C for ca. 2-4 (for log phase cultures) or 18 hrs (stationary phase cultures) using either LB or MM. All total aerobic plate counts (TAPC) were performed using the 6 × 6 drop plate method [17] with LB followed by incubation at 20-22°C (lab temperature) for 16-18 hours. Using Microsoft Excel's formulaic protocol, the TAPC-based doubling time = 1/LINEST(LOG(TAPC_1_:TAPC_n_,2),t_1_:t_n_) where the values TAPC_1 _through TAPC_n _are log-linear with respect to associated growth times t_1 _to t_n_; n was typically 6-8 points. All TAPC studies were performed using highly diluted stationary phase cells (initial colony forming unit [CFU] concentration or C_I _≥ 10^3 ^CFU mL^-1^) in either LB or MM.

### Steady State Oxygen

O_2 _levels ([O_2_], units of μM) were measured using a Clark-type oxygen electrode (Model 5300, Yellow Spring Instruments) connected to a Gilson water-jacketed chamber (1.42 mL; circulating water bath attached, 37°C) containing a magnetic stirring bar. Air-saturated 37°C water was used for calibration. To determine steady-state [O_2_] in shaking/bubbled cultures, samples were withdrawn with a syringe from bacterial culture flasks at various time points during mid-to late-log phase growth, and the oxygen consumption (e.g., [O_2_] dropping with time) determined without vortexing. The time lapse between sample withdrawal and the first [O_2_] data point was recorded and used to back-calculate the [O_2_] at the time of sampling. These same samples were then vortexed ca. 15 sec and [O_2_] measured again as a function of time. The rate of O_2 _consumption was calculated from the slope of cell density-normalized [O_2_] (TAPC plating was performed simultaneously on LB) as a function of time (apparent K_m _~ 15 ± 6 μM) [18].

### 96-well Microplate Protocol

In order to avoid water condensation which might interfere with absorbance readings, the interior surface of microplate covers were rinsed with a solution of 0.05% Triton X-100 in 20% ethanol [12] and dried in a microbiological hood under UV light. About 270 μL of each bacterial cell concentration was pipetted into every well. Each initial concentration (C_I_) is equal to C_0 _Φ_I _where C_0 _is the cell density from liquid culture (either log or stationary phase). When C_0 _≤ 10^8 ^CFU mL^-1^, the cells were sampled from an early-to mid-log phase culture. When C_0 _≥ 10^9 ^CFU mL^-1^, the cells were sampled from a stationary phase culture. Typically, each 96-well microplate contained 2 replicates each of the 8 least dilute samples (Φ = 3×10^-3 ^to 5×10^-6^; 16 wells), 4 replicates of the next 4 highest dilutions (Φ = 2×10^-6 ^to 5×10^-7^; 16 wells), 8 replicates each of the following 2 dilutions (Φ = 2×10^-7 ^or 1×10^-7^; 16 wells), and, lastly, 24 replicates of the 2 most dilute samples (Φ = 6×10^-8 ^or 3×10^-8^; 48 wells). The 96-well plate was then covered with the Triton-treated top, placed in a temperature-equilibrated Perkin-Elmer HTS 7000+ 96-well microplate reader, and monitored for optical density (OD) under the following conditions: λ = 590 nm; the time between points (Δt) = 10-25 min; total points = 50-110; temperature = 37°C; 5 sec of moderate shaking before each reading (see Results section). These dilutions, listed above, produced at least some negative (no growth) readings mainly associated with the 4 most dilute sets of wells. This lack of growth in wells associated with these dilutions is evidence for single CFU-based growth occurrences at these low C_I_. Thus, these low C_I _have been diluted to such a degree that at least an occasional random sampling of 270 μL should contain no cells at all. Generally speaking, the most probable number (single dilution MPN) calculation for these dilutions agreed with the plate count estimate. The variability of growth parameters at such low concentrations (~ 1 CFU/well) has generated much recent interest [4,6-8].

### Calculations

**Figure 8:**
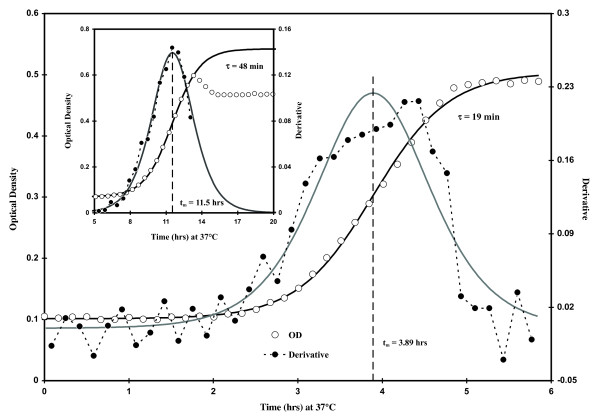
**Plot of optical density at 590 nm (open circles) and associated first derivative (ΔOD/Δt, closed circles) data associated with *E. coli *growth (C_I _~ 4,000 CFU mL^-1^) at 37°C in Luria-Bertani broth**. Inset Figure: **OD and first derivative data associated with growth (C_I _~ 7,000 CFU mL^-1^) at 37°C in a defined minimal medium (MM)**. The growth parameter, t_m_, calculated using **Eq. 1**, is shown as at the center of symmetry about the maximum in ΔOD/Δt.

After completion of any OD with time growth experiment, a tab-delimited text file was generated and data pasted into a Microsoft Excel spreadsheet formatted to display the data arrays as individual well ODs associated with each time. Typical OD growth curves are presented in Fig. 8 which have been curve-fitted (non-linear regression) to the Boltzmann equation (**Eq. 1**), a well-known sigmoidal function used in various physiological studies [19](1)

While Eq. 1 is an empirical equation, it does rely on a first order rate constant (k) therefore the doubling time can be extracted as τ = k^-1 ^Ln [2]. All curve-fitting was performed using a Gauss-Newton algorithm on an Excel spreadsheet [20]. In **Eq. 1**, OD_I _is the estimated initial optical density (0.05-0.1), OD_F _is the calculated final OD (0.5-0.7), k is the first-order rate constant, and t_m _is the time to OD_F _÷ 2. The Boltzmann relationship appears to be generally useful with optically-based growth results since excellent fits were achieved (21°C growth in LB, τ = 3.26 ± 0.0292 hrs) when **Eq. 1 **was utilized to fit previously published [21] bacterial growth data from a microchemostat.

As demonstrated previously [12], t_m _can be used (for high C_I_) as a method for estimating cell density. The inset plot in Fig. 8 shows both OD and first derivative (ΔOD/Δt) versus time data sets that were typically observed when growing our native *E. coli *isolate in MM. In order to achieve the best fit in the region which provides the most information (i.e., the exponential increase in OD), we have truncated these data and used only 2-10 points beyond the apparent t_m _to fit to **Eq. 1**. Such data abbreviation had only minor effects on the growth parameters: e.g., if the OD[t] data points in the main plot of Fig. 8 were truncated to only 3 points past the calculated t_m_, τ would change only from ~ 19.2 to 19.8 min and t_m _only by 0.7 min. All values of τ and t_m _reported herein are derived from such curve-fitting. Of course, t_m _can also be estimated from the x-axis value where the center of symmetry in ΔOD/Δt occurs (Fig. 8). We have tested two other microplate readers (Bio-Tek EL 312e and Tecan Safire II) in order to determine the variability in τ (from OD[t] data; C_I _> 1000 CFU mL^-1^) due to the devices themselves. The Perkin-Elmer instrument consistently gave the lowest τ values (τ = 18 ± 0.99 min) followed by the Bio-Tek (τ = 19 ± 1.0 min) and Tecan (τ = 21 ± 1.2 min); {Error Mean Square ÷ n} ^1/2^. = 0.42. It seems likely that the observed plate reader-associated differences in τ are due to instrument-based disparities in temperature.

During the log phase of growth [3], the rate of change in bacterial concentration with respect to time can be represented by the simple differential equation(2)

in this relation, k is a first order rate constant, t is the growth time, and C is the bacterial concentration. Upon rearrangement, integration between initial (C_I_) and final (C_F_) values of C, expressing k in terms of a doubling or generation time (τ = k^-1 ^Ln(2)) and solving for C_F _we see that(3)

where T is a time translation constant utilized to correct for the observed lag in cell growth. In our usage we assume that C_F _is the cell density at which the relationship between OD and C becomes non-linear. For our wild-type *E. coli *isolate [11] C_F _was typically about 5×10^8 ^CFU mL^-1^. Expressing **Eq. 3 **in terms of the time it takes to reach C_F _(OD ~ 0.6) we see that(4)

Since it is facile to approximate the value of t when C = C_F _÷ 2 and t = t_m _(Fig. 8), we have chosen to express **Eq. 4 **in terms of t_m_; making this alteration, substituting C_0_Φ_I _for C_I _and rearrangement gives(5)

**Figure 9:**
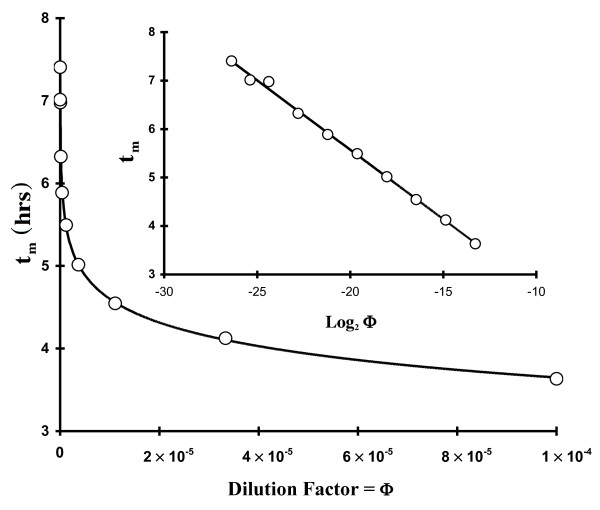
**Typical t_m _results showing its relationship (Eq. 5) with solution dilution factors (Φ) on both linear and semi-log scales**. The |slope| of the line shown in the inset figure is equal to Φ (= 0.286 hrs or 17.2 min). The parameter t_m _was calculated by fitting OD[t] data to **Eq. 1**.

In **Eq. 5 **Φ_I _is the dilution factor (e.g., for a C_I _resulting from two 1:10 dilutions Φ_I _= 0.1 × 0.1 = 10^-2^) and C_0 _is the starting cell density (e.g., from either a mid-log or stationary phase suspension of cells) from which all dilutions are made. In this work C_0 _was either about 10^8 ^(cells sampled from a mid-log phase culture; media-corrected OD_590-600 _< 0.1) or 10^9 ^(stationary phase) CFU mL^-1^. **Eq. 5 **implies that τ can be determined by calculating the slope from a plot of t_m _versus Log_2 _[Φ_I_] (Excel τ = ABS (LINEST(t_m,1 _: t_m,n_, LOG(Φ_I,1 _: Φ_I,n,2_)))). Fig. 9 displays both linear and semi-log plots of typical t_m _data plotted as a function of Φ_I_. Of course, identical results to the above are obtained if C_I _replaces C_0_Φ_I _(i.e., **Eq. 5 **with C_0 _deleted and C_I _substituted for Φ_I_)(6)

and a plot of t_m _with Log_2 _[CI] is linear (Excel τ = ABS (LINEST(t_m,1_:t_m,n_, LOG(C_I,1_:C_I,n_,2)))) with a slope equal to -τ and an intercept of (T + Log_2 _[C_F_/2]).

**Eq. 6 **implies that the time in lag phase (T) can be obtained knowing τ, C_F_, and the intercept from a plot of t_m _as a function of Log_2 _[C_I_]. When numerous values of t_m _are plotted against C_I _(semi-log plot shown in Fig. 3) by diluting either log or stationary phase cells in LB one sees a significant perturbation in T (offsets in the intercept) of the semi-log plots (10^2 ^< C_I _< 10^7 ^CFU mL^-1 ^region only). T calculations (Eq. 6) from the growth of stationary phase-diluted cells (T = 41 ± 8.4 min; average of 10 experiments; C_I _> 10^2 ^CFU mL^-1^) indicate that T was similar to lag times calculated from TAPC experiments (63 ± 9 min; average of 7 experiments). However, T values calculated in a similar fashion from log phase-diluted cells produced near-zero values (T = -11 ± 15 min; average of 8 experiments; C_I _> 100 CFU mL^-1^). Thus, the total offset between log and stationary phase-derived cells shown in Fig. 3 was about 52 min and implies that stationary phase cells require about an hour to revert to log-phase. However, because of the variability in the intercept and C_F_, we believe that the value of T using **Eq. 6 **has only a relative meaning. In other words, **Eqs. 5 **&**6 **show that variability in t_m _can be due to either variability in T, τ or both.

In order to generate the frequency of occurrence of τ values (obtained using **Eq. 1**), we first created integers from the individual τ values, counted the number of occurrences of each τ then divided this by the total number counted. Thereafter a Gaussian or normal distribution function was used to curve-fit [20] frequency of occurrence of τ data to the individually-observed τ integers. The bimodal form consisted of the sum of two Gaussians (Eq. 7) whereupon α + β = 1(7)

In **Eq. 7**, α is the fraction of the population associated with mean μ_τ1 _and standard deviation σ_τ1_; a second Gaussian is characterized by β (= 1 - α), μ_τ2_, and σ_τ2_.

Regarding other statistical methods used in this work: analysis of variance tables were generated using Microsoft Excel and standard statistical formulae for a randomized complete block design. Values for F were taken from a college-level statistics table of F-values.

## Authors' contributions

PI designed all of the experiments, performed all calculations and statistical analyses, participated in running most of the experiments and drafting the manuscript. LN carried out all the TAPC and O_2 _electrode experiments and participated in drafting the manuscript. GP and CC assisted in the experiments using conditioned media, MM, and LB with disrupted cells and participated in O_2 _electrode experiments as well as drafting the manuscript. All authors read and approved the final manuscript.
